# Identifying children who are susceptible to dropping out from physical activity and sport: a cross-sectional study

**DOI:** 10.1590/1516-3180.2018.0333050719

**Published:** 2019-10-31

**Authors:** Danilo Rodrigues Pereira da Silva, André Oliveira Werneck, Paul Collings, Rômulo Araújo Fernandes, Enio Ricardo Vaz Ronque, Luís Bettencourt Sardinha, Edilson Serpeloni Cyrino

**Affiliations:** I PhD. Adjunct Professor, Department of Physical Education, Universidade Federal de Sergipe (UFS), São Cristóvão (SE), Brazil.; II BSc. Master’s Student, Department of Physical Education, Universidade Estadual Paulista “Júlio de Mesquita Filho” (UNESP), Presidente Prudente (SP), Brazil.; III PhD. Research Fellow, Bradford Institute for Health Research, Bradford NHS Foundation Trust, Bradford, United Kingdom.; IV PhD. Full Professor, Scientific Research Group Relating to Physical Activity (GICRAF), Laboratory of Exercise Investigation (LIVE), Department of Physical Education, Universidade Estadual Paulista (UNESP), Presidente Prudente (SP), Brazil.; V PhD. Full Professor, Physical Activity and Health Laboratory, Department of Physical Education, Londrina State University, Londrina (PR), Brazil.; VI PhD. Full Professor, Exercise and Health Laboratory, CIPER, Faculdade de Motricidade Humana, Universidade de Lisboa, Lisboa, Portugal.; VII PhD. Full Professor, Study and Research Group on Metabolism, Nutrition and Exercise (GEPEMENE), Universidade Estadual de Londrina (UEL), Londrina (PR), Brazil.

**Keywords:** Adolescent, Exercise, Environment, Public health.

## Abstract

**BACKGROUND:**

Although the benefits of physical activity are clear, adherence to physical activity programs is a challenge, especially during transitional phases of life.

**OBJECTIVE:**

We aimed to identify adolescents who were more likely to drop out from physical activity and sports participation, from childhood to adolescence.

**DESIGN AND SETTING:**

This was a cross-sectional study on retrospective data regarding childhood activity among 803 Brazilian adolescents. The study was conducted at public schools in Londrina, Paraná, in 2011.

**METHODS:**

Habitual physical activity, sports participation during childhood, parental physical activity, socioeconomic status and perception of social relationships were self-reported. Cardiorespiratory fitness was estimated via a 20-m shuttle-run test and somatic maturation was estimated from the age at peak height velocity.

**RESULTS:**

Our results provided evidence that girls (physical activity: odds ratio, OR: 4.37 [95% confidence interval, CI: 1.86-10.3]; sports: OR: 2.65 [95% CI: 1.39-5.05]) and adolescents with low cardiorespiratory fitness (physical activity: OR: 1.77 [95% CI: 1.13-2.78]; sports: OR: 1.62 [95% CI: 1.15-2.26]) were more likely to drop out from active behaviors. Children with inactive mothers and inactive fathers (OR: 3.55 [95% CI: 1.12-11.3]) also showed a higher dropout rate from physical activity. Adolescents with negative perceptions of friendships (OR: 2.33 [95% CI: 1.21-4.47]) were more likely to drop out from sports.

**CONCLUSIONS:**

Higher dropout rates from active lifestyles during childhood were observed among girls and adolescents with low cardiorespiratory fitness. Parental inactivity and negative perceptions of friendships were also potential risk factors for discontinuation of childhood physical activity and sports.

## INTRODUCTION

Physical inactivity is one of the greatest causes of death worldwide.^1^ It has been estimated that if the population worldwide increased its physical activity levels, global life expectancy would increase by at least one year.^1^ However, the prevalence of physical inactivity is still high.^2^

Ecological approaches have proposed several levels of behavioral correlates, such as the political, social and built environments and intrapersonal characteristics.^3^ In this regard, early experiences with physical activity seem to be important for adoption of active lifestyles in adulthood.^4^ Additionally, among the contexts and types of physical activity, sports practices are one of the most common manifestations of active lifestyles during childhood, and this has been correlated with health in adulthood, regardless of other forms of exercise practice and daily physical activity.^5^

Although correlates of physical activity have been widely investigated in relation to static points and behavior tracking, it seems clear that active behaviors decrease during the transition from early ages.^6^ Social support through parents and friends can stimulate physical activity practice among adolescents in different domains.^7^^,^^8^ Moreover, intrinsic biological characteristics such as sex, biological maturation, nutritional status and cardiorespiratory fitness seem to be related to active behaviors.^8^^,^^9^^,^^10^ However, the correlates of dropping out from physical activity and sports, from childhood to adolescence, are still not clear.

Thus, considering that maintenance of physical activity^11^ and sports practice^12^ are protective against the short and long-term risks of cardiovascular diseases, understanding the correlates of dropping out from these behaviors during specific periods of life could help towards targeting interventions to promote sustainable active lifestyles. Kwon et al.^13^ found that adolescents with high socioeconomic status and those whose parents were active were more likely to be consistently active. Nonetheless, investigation of a greater range of variables that influence the dropout rates from physical activity and sports practice is still lacking. Such investigations could help in identifying population subgroups that would be more likely to drop out from active behavior. These subgroups would thus become targets for physical activity interventions starting at an early age. In addition, although behavioral correlates tend to vary according to social and cultural norms, no studies aimed towards identifying such groups have been conducted in low to middle-income countries, to our knowledge.

## OBJECTIVE

Thus, our aim was to analyze the psychological, biological and social correlates of dropping out from physical activity and sports practice, from childhood to adolescence, among Brazilian adolescents. Our hypothesis was that biological factors would be strongly associated with dropping out from active behaviors, and that psychological and social factors might explain part of this negative outcome.

## METHODS

### Ethical statement

The authors declare that they did not have any conflict of interests regarding the publication of this paper. This work was supported by the Brazilian Council for Scientific and Technological Development (CNPq/Brazil; procedural no. 15608/2011). The local ethics committee approved all the study procedures (CAAE: 0142.0.268.000-11), and these complied with the principles of the Declaration of Helsinki.

### Design, sample size and participants

This was a cross-sectional study conducted in 2011 among adolescents aged between 10 and 17 years old who were enrolled in public schools in Londrina, Brazil. This city had 506,701 inhabitants, a human development index of 0.778 and a gross domestic product per capita of US$ 8,530.77.^14^

The current study formed part of a project under the title “Prevalence of metabolic syndrome and cardiovascular risk factors in adolescents in Londrina.” For this project, the sample size calculation was based on the following parameters: prevalence of metabolic syndrome of 4%; α of 0.05; margin of error of two percentage points; and design effect of 2.0. This calculation indicated that at least 900 adolescents should be selected.

Recruitment of participants was performed in two stages. Initially, all the public schools in the city were categorized according to the regions of the city (north, south, east, west and center). Thereafter, two schools were randomly selected from each region. Classes within the schools chosen were then randomly selected, and all students within these classes were invited to participate in the study. Students using prescription medicines or undergoing treatment for an illness, and those who did not return the consent form with a parent’s signature, were excluded from the study.

In total, 1,395 adolescents were enrolled in the study. However, given that our aim was to analyze factors associated with dropping out from sports and physical activity from childhood to adolescence, we excluded participants who were older than 17 years (n = 17), those who had not participated in regular sports during childhood (n = 383) and those for whom physical activity data were missing (n = 192).

### Organized sports participation during childhood

To assess childhood sports participation, adolescents were asked (yes/no) if they had participated in supervised sports for at least one year between the ages of 7 and 10 (intraclass correlation coefficient, ICC = 0.87). As described above, participants who had not taken part in organized sports were excluded.

### Physical activity and organized sports participation during adolescence

The Baecke questionnaire^15^ was self-completed by the adolescents and was used as an indicator of concurrent physical activity level. This instrument contains questions relating to physical activity performed at school, during leisure time and as part of sport. The sum of all its domains constitutes the estimated habitual physical activity (through a specific score).^8^ For quality control, the questionnaire was applied again to a representative portion of the sample (10%) after an interval of seven days. The ICC for this was calculated to be 0.73.

In deriving the outcomes for this investigation, participants who were in the highest quartile of habitual physical activity (all domains) during adolescence, or those who were in the highest quartile for sports participation, were considered to have been successful in maintaining their activity behaviors. The participants were thus distributed between four non-exclusive categories:

1. Maintenance of activity (sports participation during childhood and habitually active during adolescence);
2. Maintenance of sports participation (sports participation during both childhood and adolescence);
3. Dropping out from physical activity (sports participation during childhood, but habitually inactive during adolescence;
4. Dropping out from sports participation (sports participation during childhood but not during adolescence).

### Self-perception of social relationships

Participants’ self-perception about their relationships with friends and classmates was assessed on a four-point scale ranging from “very unsatisfied” to “very satisfied” (ICC for this question = 0.50). Participants who were either “very unsatisfied” or “unsatisfied” were deemed to have a negative perception of their relationships with friends.

### Biological variables

Somatic maturation was estimated using the peak height velocity (PHV)^16^. The “distance from PHV” (how many years left until PHV) was calculated from information on height, trunk-cephalic height and leg length. After this, PHV was subtracted from chronological age, creating the age of peak height velocity. Cardiorespiratory fitness (CRF) was assessed by means of the 20-meter shuttle-run test, as designed by Leger and Lambert.^17^ This test was conducted in a multi-sports indoor court. Based on the testing time, peak VO_2_ in ­ml/­kg/­min (maximal volume of oxygen consumption) was calculated in accordance with the equation proposed by Leger et al.^18^ Low cardiorespiratory fitness was defined using the cutoff points of Fitnessgram.^19^ Body mass index (BMI) was calculated from body weight and height, which were measured using standard procedures. The technical error of measurement for these measurements was: weight = 0.68% and height = 0.37%. BMI data were categorized using the cutoffs described by Cole et al.^20^

### Parent-reported variables

The subjects’ parents provided information about their own physical activity level and the socioeconomic status (SES) of the family. The adolescents handed the questionnaires to their parents and subsequently returned them to the research team. The Baecke questionnaire^15^ was administered to provide an estimate of paternal and maternal physical activity. Parents who reported having at least 180 minutes/week of moderate to vigorous physical activity, over the previous nine months, were classified as “active”.^5^ The Brazilian Economic Classiﬁcation Criteria instrument^21^ was used to classify families into one of five SES groups ranging from A (highest SES) to E (lowest SES), based on family possessions and the educational level of the head of the household. For this study, groups D and E were considered indicative of low SES.

### Statistical analysis

Descriptive statistics (means, standard deviations and frequencies) were used to characterize the sample. Group comparisons between participants who remained physically active or remained involved in sports versus those who dropped out were performed using the Mann-Whitney and chi-square tests. Crude and adjusted logistic regression models were specified to investigate factors associated with increased likelihood of dropping out from physical activity or sports participation. Factors showing some evidence of an association with the outcome in crude analyses (P < 0.2) were taken forward for inclusion in the adjusted model and were maintained if they showed P < 0.05 in the final model. All analyses were conducted in STATA 13.0.

## RESULTS

The final sample included 803 adolescents (49.9% girls), with a mean chronological age of 12.9 ± 1.5 years. Among these subjects, 33.3% presented low socioeconomic status. In general, the adolescents who dropped out of physical activity showed lower values for age at peak height velocity and cardiorespiratory fitness. The majority of these subjects were girls, and they had inactive mothers. Likewise, the adolescents who dropped out of sports also presented lower values for age at peak height velocity and cardiorespiratory fitness (Table 1), were mostly girls and also reported negative perceptions of friendships.

**Table 1.** Descriptive statistics and comparison of independent variables between physical activity and sports participation “dropouts” versus “maintainers”, from childhood to adolescence

	Physical activity		P	Sports participation		P
	Dropouts (n = 536)	Maintainers (n = 267)		Dropouts (n = 543)	Maintainers (n = 260)	
Sex (girls)	60% (55.3 to 63.6)	31% (25.5 to 36.5)	< 0.001	58% (54.0 to 62.3)	33% (27.3 to 38.6)	< 0.001
Chronological age (years)	13 ± 1.61	13 ± 1.46	0.661	13 ± 1.58	13 ± 1.48	0.544
Age at PHV (years)	13 ± 1.22	14 ± 1.14	< 0.001	13 ± 1.21	14 ± 1.17	< 0.001
BMI (kg/m^2^)	19.8 ± 3.65	20.1 ± 1.13	0.450	19.9 ± 4.02	20.0 ± 3.96	0.549
CRF (ml/kg/min)	39.7 ± 4.71	42.1 ± 4.85	< 0.001	39.8 ± 4.68	42.1 ± 4.96	< 0.001
No sports participation	89% (86.1 to 91.4)	24% (19.9 to 30.2)	< 0.001	100% (98.3 to 100)	0% (0 to 0.9)	< 0.001
Inactive	100% (98.3 to 100)	0% (0 to 0.9)	< 0.001	22.7% (18.0 to 28.2)	87.8% (84.8 to 90.3)	< 0.001
Overweight (BMI)	23% (19.8 to 27.1)	19% (14.5 to 24.2)	0.171	23% (19.7 to 26.9)	19% (14.7 to 24.5)	0.214
Low CRF	54% (49.8 to 58.7)	42% (35.6 to 47.9)	0.001	54% (49.7 to 58.4)	41% (35.1 to 47.8)	0.001
Low SES	32% (27.4 to 36.4)	37% (30.6 to 44.2)	0.187	32% (27.9 to 36.8)	36% (29.8 to 43.5)	0.309
Inactive mother	97% (95.4 to 98.7)	92% (87.1 to 95.4)	0.003	96% (93.9 to 97.8)	95% (90.4 to 97.4)	0.447
Inactive father	94% (91.4 to 96.5)	90% (84.2 to 93.8)	0.066	93% (88.3 to 96.6)	93% (89.6 to 95.1)	0.793
Friends*	10% (7.7 to 12.8)	7% (4.9 to 11.4)	0.262	11% (8.8 to 14.1)	5% (4.7 to 13.5)	0.005

Data are presented as mean ± standard deviation or % (with 95% confidence intervals). PHV = peak height velocity; BMI = body mass index; CRF = cardiorespiratory fitness; SES = socioeconomic status; *Negative perceptions of relationships with friends.

**Figure 1.**
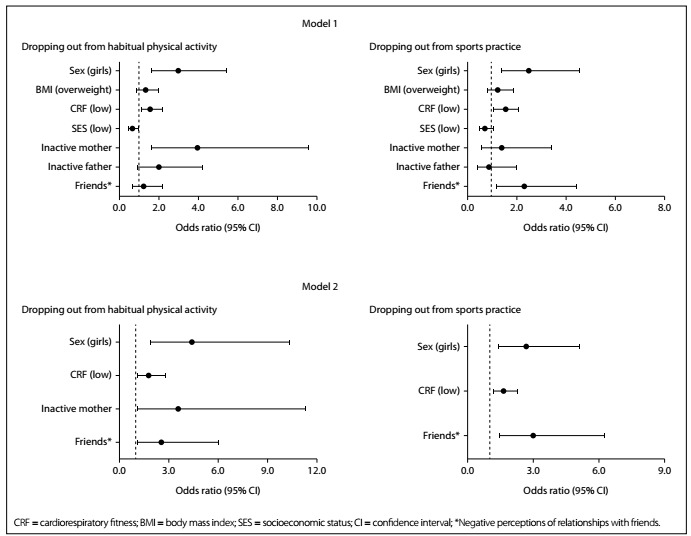
Correlates of dropping out from physical activity and sports participation, from childhood to adolescence. Model 1 = unadjusted crude associations. Model 2 = adjusted for adolescent age, age at peak height velocity and all other variables in the model.

Figure 1 shows the odds ratios for dropping out from physical activity and sports participation. The first (unadjusted) model showed that girls, individuals with low cardiorespiratory fitness and children with inactive mothers were more likely to drop out of physical activity. In the unadjusted model, children from low SES families were less likely to drop out from physical activity. However, in the second (adjusted) model, SES ceased to be significantly related to dropping out from physical activity. In the second model, paternal physical activity emerged as an additional factor that was associated with increased likelihood of dropping out.

Girls and any adolescents with low cardiorespiratory fitness or negative perceptions of friendships were more likely to drop out from participating in sports. The associations for dropping out from sports remained unchanged following adjustment of model 2 for the adolescents’ ages and their ages at PHV.

## DISCUSSION

This was a cross-sectional study that used retrospective information relating to early sports participation. Our aim was to identify adolescents who were more likely to drop out from active behaviors, from childhood to adolescence, by analyzing correlates of dropping out from physical activity and sports practice. We found that girls and any adolescents who had low cardiorespiratory fitness were more likely to drop out from both sports participation and habitual physical activity (all domains). Moreover, adolescents who had two inactive parents and who reported having negative perceptions of friendships exhibited, respectively, greater frequency of dropping out from habitual physical activity and from sports practice.

Physical inactivity is associated with several chronic diseases and metabolic risk factors.^22^ Sports participation during childhood seems to be a protective factor for cardiovascular risk in adulthood.^5^ Several paths have been proposed to explain the influence of early sports participation on health in adulthood.^23^ Among these, maintenance of active behaviors (sports/physical activity) at subsequent ages and their positive effects on body fat regulation, as well as more direct paths through DNA methylation,^23^^,^^24^ have been highlighted. However, it seems clear that physical activity decreases over individuals’ lifetimes.^6^^,^^25^ Thus, although correlates of physical activity at static points have frequently been investigated,^3^ less attention has been given to correlates of longitudinal changes in behavior.

In the present study, we found that girls were more likely to drop out from both physical activity and sports participation. One possible explanation for this finding may be that boys are given greater encouragement to perform physical exercise during leisure time.^10^ This may be one of the reasons for the lower prevalence of physical activity among girls. It was thus expected that there would be a greater likelihood that girls would drop out. Furthermore, biological maturation is an important confounder in analyses on physical activity among girls because of differences in their intrinsic characteristics, such as increasing amounts of body fat and psychological variables.^26^ However, even after adjustment for somatic maturation, we observed that girls were more likely to drop out from sports practice and habitual physical activity.

These findings indicate that social issues may have more longitudinal influence on sports participation and general physical activity than biological factors.^27^ It is well established that women’s social roles (e.g. the notions of being fragile or delicate, or of being housewives) are not associated with physical effort or physical activity and sports practice. Thus, during this transitional phase (adolescence), girls tend to be less encouraged by their parents and friends to be physically active. This finding extends the discussion in the current literature on the perspective of dropping out from active behaviors.

Similarly, the adolescents who presented low cardiorespiratory fitness showed greater likelihood of dropping out from both of these active behaviors. Given that physical inactivity is negatively associated with cardiorespiratory fitness,^28^ adolescents who drop out from physical activity or sports practice tend to present lower values for cardiorespiratory fitness. On the other hand, adolescents with lower physical fitness also tend to adopt a less active lifestyle.^29^ Because of the study design, and since we had no early information on or measurement of cardiorespiratory fitness, our findings tend to support the first hypothesis.

Interestingly, we found that inactivity among parents was associated with their offspring’s dropping out from physical activity, but not from sports practice. This result may have been be due to transference of behaviors from parents to their children,^30^ since inactive lifestyles among parents could influence their children to be less active (overall indicator). Thus, inactive parents can still support their offspring’s sports practice.^31^ However, sports practice seems to be more influenced by friends and characteristics of intrinsic motivation.^7^ In this regard, our results provide support for the notion that social relationships have a role in maintenance of health-related behaviors, since we found that negative perceptions of friendships were related to dropping out from sports participation. Previous studies also demonstrated that perceptions of social relationships are correlated with leisure-time physical activity and sports practice,^7^^,^^8^ especially regarding collective forms of sports activities.^32^

While low socioeconomic status is an important issue in terms of healthcare, we found that it seemed to protect against dropping out from physical activity in the crude analysis. Adolescents with lower socioeconomic status, especially in developing countries, are more active in the domains of transportation and occupation and, consequently, tend to have a more active lifestyle.^33^^,^^34^ However, no association was found between socioeconomic status and dropping out from sports practice. It is important to note that our sample was relatively homogeneous regarding socioeconomic status (recruitment from public schools), which could explain the lack of significance of this variable in the final model for dropping out from physical activity. However, the parents’ physical activity may have overlapped with this effect (Figure 1; Model 2), since socioeconomic status is an important predictor of physical activity in adulthood.^33^

The results from the present study lead to some practical applications. Firstly, special attention could be given to girls and to adolescents with low cardiorespiratory fitness. Sustainable early interventions should be conducted in these specific target groups, in order to promote adequate levels of physical fitness during adolescence.^35^ Moreover, bearing in mind that physical inactivity among parents is related to adolescents’ dropping out from physical activity, family-based interventions could be conducted to increase the levels of physical activity among both parents and adolescents, as well as to improve parental social support for adolescents. School-based interventions that promote good social interactions between children should also be encouraged.

Concerning our methods, this study had the limitation that only one physical activity domain during childhood (supervised sports practice) was assessed. Even though supervised sports practice is the greatest manifestation of physical activity during this phase^36^ there was the potential for recall bias, caused by the retrospective design of our study. We did not take into consideration unsupervised sports participation.

Moreover, our measurement of physical activity and sports practice during adolescence was self-reported. Nonetheless, the physical activity questionnaire showed good reproducibility (ICC = 0.73). Similarly, physical activity among the participants’ parents was also self-reported, and thus could also have presented bias. The lack of reproducibility of the information in the parents’ questionnaire is an important limitation.

Lastly, because of the study design (cross-sectional), we were unable to discern the temporality/causality between the variables. Nevertheless, these initial data can provide support for future longitudinal studies.

Among the strengths of this study, it was the first to evaluate correlates of dropping out from physical activity and sports practice, from childhood to adolescence, in Brazil. We can highlight the control over biological maturation in the adjusted analyses: this is an important confounding variable in physical activity studies conducted among adolescents.^9^ Our study presented correlates of dropping out from physical activity and sports practice that were derived from data on more than 800 adolescents from a middle-income country of continental dimensions and distinct culture.

## CONCLUSIONS

Girls and adolescents who presented low cardiorespiratory fitness were more likely to drop out from active behaviors. Parents’ physical inactivity and negative perceptions of friendships among the students were, respectively, also related to increased rates of dropping out from physical activity and sports practice, from childhood to adolescence.
